# Multiple sexual partnerships and associated factors among young psychoactive-substance-users in informal settlements in Kampala, Uganda

**DOI:** 10.1371/journal.pone.0239323

**Published:** 2020-10-06

**Authors:** Tonny Ssekamatte, Moses Tetui, Simon P. S. Kibira, John Bosco Isunju, Richard K. Mugambe, Elizabeth Nabiwemba, Solomon Tsebeni Wafula, Esther Buregyeya, Justine Nnakate Bukenya

**Affiliations:** 1 Department of Disease Control and Environmental Health, Makerere University School of Public Health, Umeå, Sweden; 2 Department of Health Policy and Planning, Makerere University School of Public Health, Umeå, Sweden; 3 Département of Epidemiology and Global Health, Umeå University, Umeå, Sweden; 4 Department of Community Health and Behavioural Sciences, Makerere University School of Public Health, Umeå, Sweden; Qazvin University of Medical Sciences, ISLAMIC REPUBLIC OF IRAN

## Abstract

**Background:**

Multiple sexual partnerships increase the risk of transmission of HIV and can be exacerbated by substance abuse. However, the association between psychoactive substance use and multiple sexual partnerships among young people in informal settlements of low-income countries is not well known. This study established the prevalence of multiple sexual partnerships and associated factors among young psychoactive-substance-users in informal settlements in Kampala, Uganda.

**Methods:**

This was a cross-sectional study involving 744 young (aged 18–24 years), sexually active, psychoactive substance-users selected from 12 of the 57 informal settlements of Kampala City. The prevalence of multiple sexual partnerships and their differential distribution by socio-demographic strata was established. Modified Poisson regression models were run in Stata 14 software to generate prevalence rate ratios for the factors associated with multiple sexual partnerships.

**Results:**

About 40.6% (37.9% of males and 50.0% of females) had engaged in multiple sexual partnerships in the last 30 days. Engaging in multiple sexual partnerships in the last 30 days was positively associated with being female (PR 1.29, 95% CI: 1.03–1.63); staying in the informal settlement for 6–10 years (PR 1.34, 95% CI: 1.02–1.75) and chewing khat in the last 30 days (PR 1.93, 95% CI: 1.10–3.40).

**Conclusion:**

Multiple sexual partnerships are highly prevalent among young psychoactive-substance-users, irrespective of the socio-demographic strata. Being female, having lived in the informal settlement for 6–10 years, and chewing khat were significantly associated with having multiple sexual partners in the last 30 days. In tackling this high-risk sexual behaviour, it is recommended that risk-reduction interventions are considered for the different socio-demographic strata identified in this study, i.e. females, those who have lived in the informal settlement for about 6–10 years, and those who chew khat.

## Background

Globally, meeting the sexual and reproductive health needs of young people remains a public health challenge [1,2]. Thus, negative sexual and reproductive health outcomes remain prevalent. For instance, in 2016, UNAIDS reported over 620,000 new HIV infections among young people aged 15–24 years [3]. These infections are indicative of a high prevalence of high-risk sexual behaviours such as inconsistent condom use, having sexual intercourse under the influence of psychoactive substances and multiple sexual partnerships [4,5], and cross-generational sex.

Young psychoactive-substance-users include individuals aged 10–24 years who use substances such as alcohol, khat, marijuana, heroin, and kuba. Such substances have the ability to impair the user’s cognition and decision making [6], thereby increasing their risk of having unsafe sexual intercourse. There is also evidence of young psychoactive-substance-users having a higher likelihood of inconsistent condom use and multiple sexual partnerships compared to non-users [7–12].

Multiple sexual partnerships or relationships, defined as having more than one sexual partner over a specified period of time, remain crucial in the transmission of Sexually Transmitted Infections (STIs) [13,14]. Multiple sexual partners could either be serialized or concurrent [15]. These partnerships are highly prevalent among young people in Uganda, especially those residing in informal settlements [12,16]. Engaging in multiple sexual partnerships is often driven by the desire for increased sexual pleasure, cultural norms, partner infidelity, and economic necessity [17–20].

The classification of multiple sexual partnerships as a high-risk sexual behaviour remains contentious [21]. Different scholars have argued that studies examining multiple sexual partnerships as a high-risk sexual behaviour do not take into account the sexually transmitted disease (STD) status, and consistent condom use among multiple partners [22,23]. Therefore, some authors argue that such studies are likely to overestimate the prevalence of high-risk sexual behavior [22]. However, this does not disregard the significance of undertaking such studies. Our study is premised on the precautionary principle in that understanding the prevalence of multiple sexual partnerships irrespective of condom use at last sexual intercourse and the STD status among multiple sex partners is vital in characterising high-risk sexual behaviours in informal settlements. After all, young psychoactive-substance-users in informal settlements are at an elevated risk of STIs, including HIV, compared to other urban, and rural residents in non-informal settlements [24]. Additionally, the high prevalence of HIV and commercial sex in informal settlements in Kampala implies an elevated risk of transmission of HIV, particularly among young psychoactive-substance-users [25].

Informal settlements are unplanned residential areas mainly habituated by the urban poor, whose housing is often not in compliance with up-to-date planning and building regulations [26]. These are also characterised by congestion and a lack of access to basic services and infrastructure including healthcare [27]. The informal settlements of Kampala are a closed community, with a high likelihood of the dwellers, including young psychoactive-substance-users engaging in unsafe multiple sexual relationships with sex workers, and HIV-infected individuals within their circles [16,28]. Unprotected sexual intercourse increases opportunities for transmission of STIs [13,29–32]. Despite these risk factors, little is known about the magnitude and factors associated with multiple sexual partnerships among young psychoactive users.

Given the behavioural and social vulnerability of young psychoactive-substance-users, focussing on multiple sexual partnerships provides a reliable indicator for surveillance of STIs in informal settlements [4,5]. This study used the social exchange theory [33–35] to establish the prevalence of multiple sexual partnerships across socio-demographic strata, and associated factors among young psychoactive-substance-users in the informal settlements of Kampala. Based on the social exchange theory, young psychoactive-substance-users engage in multiple sexual partnerships with the anticipation of a reward or return from the recipients of the gift, which in this case is sexual intercourse. The social exchange theory has previously been used in various studies [12,36,37] to understand the drivers of multiple sexual partnerships. Findings from this study can be used to inform the design of appropriate STI prevention interventions among young people, particularly those who use psychoactive substances.

## Methods

### Study design, setting, and respondents

This cross-sectional study was conducted in the informal settlements in Kampala, Uganda’s capital city, from June to July 2019. Kampala city has a population of over 1,507,080 people, among whom 27.5% are aged 15–24 years and 15.8% are aged 10–17 years. Kampala city occupies approximately 189 Km^2^, and is Uganda’s main economic hub, generating 65% of the national GDP [38]. Data were collected from 4 of the 5 divisions: Kawempe, Makindye, Lubaga, and Nakawa divisions. These divisions have the largest number of informal settlements, and young psychoactive-substance-users. Only young psychoactive-substance-users aged between 18 and 24 years and who had been residing within the informal settlements of Kampala city for at least 6 months prior to the survey were included in the survey. The age group of 18–24 years was chosen because it is above the legal age and such young people have the autonomy to engage in psychoactive substance use without parental restrictions or consent. In terms of eligibility, research assistants only interviewed respondents who were in a good mental state, and were not sick at the time of the survey.

### Sample size and sampling

A minimum sample size of 770 respondents was determined using the Kish Leslie formula for cross-sectional studies [39]. We considered a conservative prevalence of multiple sexual partnerships among young psychoactive-substance-users of 50.0%, a margin of error (d) of 5% corresponding to a 95% level of confidence and a design effect of 2.0 [40]. This yielded a sample size of 770 respondents.

Twelve out of fifty-seven informal settlements were purposively selected from a list of informal settlements obtained from the Kampala Capital City Authority (KCCA) Department of Public Health Services and Environment (4 in Nakawa; 4 in Kawempe, 2 in Lubaga, and 2 in Makindye divisions). These informal settlements were purposively selected for geographical representativeness of the informal settlements in the entire city. Besides, the settlements also had the highest average house hold size. The informal settlements included Kinawataka, Luzira, Luzira-Kirombe, Kitintale, Nalukolongo, Wankulukuku-Kabowa, Kamwokya, Bwaise, Katanga, Katwe-Kinyoro, Namuwongo-Soweto and Kyebando. We used respondent-driven sampling (RDS), a form of chain-referral network sampling, to select respondents. This approach is recommended for hard-to-reach populations such as psychoactive substance users in informal settlements [40].

In this study, the research assistants enrolled 4 psychoactive substance users (both male and female) from each informal settlement as ‘seeds’ using contacts obtained in an earlier study [41]. These seeds were not under the influence of any psychoactive substance at enrolment. Consenting seeds were first interviewed and then given 3 predetermined coupons and briefly trained on how to recruit their peers into the study. Coupons contained information about the study and its aim, coupon start and expiry date, coupon identification number, survey location, contact details of the principal investigator, and the hours of operation. The expiry dates helped research assistants to compute the valid and expired coupons still in circulation. Nonetheless, the enrolment of respondents presenting with expired coupons was allowed as long as they met the eligibility criteria. The primary seeds then asked the secondary seeds to report for the interview. These respondents constituted the first wave. In turn, their recruits who then participated in the survey formed the second wave. Interviews with the different seeds were conducted at appropriate times (9:00am– 5:00am) from static convenient and appropriate locations (with privacy and relatively quiet) such as restaurants and bars. This was continued until the survey achieved the minimum sample size.

### Data collection

Data were collected between June and July 2019 using a structured questionnaire. The questions used in the study tool were adopted from the Uganda Demographic Health Survey and the Global School-based Student Health Survey tools. These tools are arleady validated, and are used to collect information on high-risk sexual behaviours and alcohol consumption [42,43]. The study questionnaire was uploaded to the KoBoCollect mobile application and administered via handheld mobile devices (Android phones and tablets). The tool was pretested among 20 psychoactive substance users in an informal settlement in Kajjansi Town Council, Wakiso district, and relevant adjustments were made. Respondents were interviewed by eight trained research assistants. The research assistants had a minimum of a Bachelor’s degree in health sciences or humanities.

Data collected included: socio-demographic characteristics such as age, educational status, marital status, average monthly income, duration of stay in the informal settlement, and history of use of a particular psychoactive substance, in addition to the number of sexual partners in the last 30 days

### Study variables

The outcome of interest was engaging in multiple sexual partnerships in the last 30 days. Respondents were asked how many sexual relationships they had in the last 30 days. Those who reported more than one sexual relationship in the target period were considered to have engaged in multiple sexual partnerships [14], irrespective of whether they were concurrent or serial. The independent variables included socio-demographic characteristics such as age, marital status, staying with or without parents, average monthly income, level of formal education, and history of use of a particular psychoactive substance.

### Statistical analyses

Descriptive statistics such as means and standard deviations were used to summarize continuous data while categorical data were expressed as frequencies and proportions. A chi-square test was performed to indicate statistically significant differences between the prevalence of multiple sexual characteristics across the sociodemographic strata and duration of use of each substance. The main outcome was dichotomized as “ever engaging in multiple sexual relationships in the last 30 days or not”. A “modified” Poisson regression analysis was applied to model the association between “ever engaging in multiple sexual partnerships in the last 30 days” and the independent variables. Simpler models consisting of the outcome and one predictor were initially run and variables with p-values less than 0.2 included in the multivariable model [44]. Prevalence Ratios (PRs) were used as measures of associations because they are more conservative measures of associations when the prevalence of the outcome of interest is greater than 10% [45]. We adjusted for known confounders such as age and sex. All analyses were done using Stata 14 (StataCorp, Texas).

### Ethics statement

Ethical approval was granted by Makerere University School of Public Health Higher Degrees and Research Ethics Committee (MakSPH HDREC). Administrative clearance to conduct this study was obtained from KCCA and the area local leaders. Informed written consent was sought from all respondents.

## Results

### Social demographic characteristics of the respondents

There were 744 sexually active young psychoactive-substance-users with a mean age of 21.5 years (SD 2.17), representing a response rate of 96.6%. About 78% of the respondents were male, and 69.1% had never been married, while 85.3% were not living with their parents/guardians (Table 1).

**Table 1:** Social demographic characteristics of sexually active young-psychoactive-substance users.

Variable	Attribute	Frequency (n = 744)	Percentage (%)
Age of participant Mean (SD) = 21.5 (2.17)	18–19	174	23.4
	20–24	570	76.6
Sex	Male	580	78.0
	Female	164	22.0
Marital status	Never married	514	69.1
	Currently married	162	21.8
	Divorced/Separated	68	9.1
Highest level of education	Primary	310	41.7
	Post primary	434	58.3
Religion	Catholic	291	39.1
	Anglican	126	16.9
	Muslim	222	29.8
	Pentecostal	80	10.8
	Other religion	25	3.4
Living with parents	Yes	109	14.7
	No	635	85.3
Duration of staying in the area (in years)	≤ 5	264	35.5
	6–10	145	19.5
	> 10	335	45.0
Average monthly income (USD) Prevailing exchange rate (1 USD = UGX 3676)	≤ 68	477	64.1
	68.1 < USD ≤136	204	27.4
	> 136	63	8.5

### Prevalence of multiple sexual partnerships

Overall, 40.6% of the respondents had engaged in multiple sexual partnerships within the last 30 days prior to the interview. Half (50.0%) of the female respondents had engaged in multiple sexual partnerships. Sex (p-value = 0.005) and the duration of stay in the informal settlements (p-value = 0.016) were significantly associated with engaging in multiple sexual relationships in the last 30 days. Forty-two per cent of the adolescents aged 18–19 years, 45.5% with no formal education and 42.9% of those earning above UGX 500,000 (≈USD 138) per month were engaged in multiple sexual relationships (Table 2).

**Table 2:** Distribution of multiple sexual partnerships based on socio-demographic characteristics.

Variable	Attribute	Engaged in multiple sexual relationships in the last 30 days		Chi-square p-value
		Yes	No	
Sex	Male	220 (37.9)	360 (62.1)	
	Female	82 (50.0)	82 (50.0)	0.005*
Age category	18–19	73 (42.0)	101 (58.0)	
	20–24	229 (40.2)	341 (59.8)	0.676
Level of education	Primary	137 (44.2)	173 (55.8)	
	Post primary	165 (38.0)	269 (62.0)	0.009*
Marital status	Never married	207 (40.3)	307 (59.7)	
	Currently married	59 (36.4)	103 (63.6)	0.064
	Divorced/Separated	36 (52.9)	32 (47.1)	
Religion	Catholic	122 (41.9)	169 (58.1)	
	Anglican	44 (34.9)	82 (65.1)	
	Muslim	92 (41.4)	130 (58.6)	
	Pentecostal	37 (46.3)	43 (53.8)	
	Other	7 (28.0)	18 (72.0)	0.323
Living with parents	Yes	43 (39.4)	66 (60.6)	
	No	259 (40.8)	376 (59.2)	0.793
Average monthly income (USD) Prevailing exchange rate (1 USD = UGX 3676)	≤ 68	194 (40.7)	283 (59.3)	
	68.1 < USD ≤136	81 (39.7)	123 (60.3)	
	> 136	27 (42.9)	36 (57.1)	0.904
Duration of stay in informal settlement	0–5 years	98 (37.1)	166 (62.9)	
	6–10 years	74 (51.0)	71 (49.0)	
	More than 10 years	130 (38.8)	205 (61.2)	0.016*

### Distribution of multiple sexual partnerships in the last 30 days based on type, and reasons for engaging in multiple sexual partnerships

Engaging in multiple sexual partnerships in the last 30 days was statistically higher among those who drunk alcohol in the last 30 days (p = 0.007), and those who chewed khat (p = 0.025).

The reasons young psychoactive-substance-users gave for engaging in multiple sexual relationships in the last 30 days were; sexual satisfaction (52.6%), pleasure (50.0%), peer pressure (26.8%) and earning money (24.8%). Of those who engaged in multiple sexual partnerships, 89% of the females and 1% of males did so because of money, 62.7% of males and 25.6% of females did so for sexual satisfaction, while 61.8% of males and 18.3% of females did so for pleasure. (Table 3).

**Table 3:** Prevalence of multiple sexual partnerships among young psychoactive-substance-users in informal settlements in Kampala.

Variable	Attribute	Engaged in multiple sexual partnerships in the last 30 days		Chi-square p value
		Yes	No	
**History of ‘ever used’ a psychoactive substance (n = 744)**				
Ever drunk alcohol	Yes	271 (43.1)	358 (56.9)	
	No	31 (27.0)	84 (73.0)	0.001*
Ever used marijuana	Yes	204 (44.1)	259 (55.9)	
	No	98 (34.9)	183 (65.1)	0.013*
Ever chewed khat	Yes	203 (42.7)	272 (57.3)	
	No	99 (36.8)	170 (63.2)	0.113
Ever used kuba	Yes	66 (55.0)	54 (45.0)	
	No	236 (37.8)	388 (62.2)	0.001*
Ever used heroin	Yes	25 (56.8)	19 (43.2)	
	No	277 (39.6)	423 (60.4)	0.024*
**History of substance use in the last 12 months**				
Drunk alcohol in the last 12 months (n = 629)	Yes	263 (43.5)	341 (56.5)	
	No	8 (32.0)	17 (68.0)	0.253
Used marijuana in the last 12 months (n = 463)	Yes	190 (44.4)	238 (55.6)	
	No	14 (40.0)	21 (60.0)	0.615
Chewed khat in the last 12 months (n = 475)	Yes	195 (43.3)	255 (56.7)	
	No	8 (32.0)	17 (68.0)	0.265
Used kuba in the last 12 months(n = 120)	Yes	54 (59.3)	37 (40.7)	
	No	12 (41.4)	17 (58.6)	0.090
Used heroin in the last 12 months (n = 44)	Yes	19 (70.4)	8 (29.6)	
	No	6 (35.3)	11 (64.7)	0.022*
**History of substance use in the last 30 days**				
Drunk alcohol in the last 30 days (n = 604)	Yes	250 (45.2)	303 (54.8)	
	No	13 (25.5)	38 (74.5)	0.007*
Used marijuana in the last 30 days (n = 428)	Yes	173 (44.8)	213 (55.2)	
	No	17 (40.5)	25 (59.5)	0.591
Used kuba in the last 30 days (n = 91)	Yes	42 (62.7)	25 (37.3)	
	No	12 (50.0)	12 (50.0)	0.278
Chewed khat in the last 30 days (n = 450)	Yes	184 (45.0)	225 (55.0)	
	No	11 (26.8)	30 (73.2)	0.025*
Used heroin in the last 30 days (n = 27)	Yes	9 (64.3)	5 (35.7)	
	No	10 (76.9)	3 (23.1)	0.472

A p-value of ≤ 0.05 at a 95% CI was considered statistically significant.

### Factors associated with multiple sexual partnerships among young psychoactive-substance-users

Female psychoactive substance users had a 29% higher likelihood of engaging in multiple sexual relationships compared to males (PR 1.29, 95% CI: 1.03–1.63, p = 0.026). Compared to respondents who had lived in informal settlements for ≤ 5 years, those who had stayed for 6–10 years had a 34% higher likelihood of engaging in multiple sexual relationships (PR 1.34, 95% CI: 1.02–1.75, p = 0.034). Young psychoactive-substance-users who had chewed khat in the last 30 days had a 93% higher likelihood of engaging in multiple sexual partnerships compared to those who had not (PR 1.93, 95% CI: 1.10–3.40, p = 0.021). At multivariable analysis, drinking alcohol, using marijuana, heroin, or kuba were not associated with engaging in multiple sexual partnerhsips. (Table 4).

**Table 4:** Factors associated with engaging in multiple sexual relationships among young psychoactive-substance-users in informal settlements in Kampala.

Variable	Freq (n)	Engaged in multiple sexual relationships in the last 30 days		Unadjusted PR (95% CI)	Adjusted PR (95% CI)	P value
		Yes n (%)	No n (%)			
**Sex**						
Male	580	220 (72.8)	360 (81.4)	1	1	
Female	164	82 (27.2)	82 (18.6)	1.31 (1.09–1.58)	1.29 (1.03–1.63)	0.026*
**Age category**						
18–19	174	73 (24.2)	101 (22.9)	1	1	
20–24	570	229 (75.8)	341 (77.1)	0.95 (0.78–1.17)	0.93 (0.73–1.20)	0.613
**Marital status**						
Never married	514	207 (68.5)	307 (59.7)	1	1	
Currently married	162	59 (19.5)	103 (63.7)	0.90 (0.71–1.13)	0.88 (0.65–1.18)	0.415
Divorced/separated	68	36 (11.9)	32 (47.1)	1.31 (1.02–1.68)	1.07 (0.79–1.47)	0.636
**Living with parents**						
Yes	109	43 (14.2)	66 (14.9)	1		
No	635	259 (85.8)	376 (85.1)	1.03 (0.80–1.32)		
**Average monthly income**						
≤ 68	477	194 (64.2)	283 (64.0)	1		
68.1 < USD ≤136	204	81 (26.8)	123 (27.8)	0.97 (0.79–1.19)		
> 136	63	27 (9.0)	36 (8.2)	1.05 (0.77–1.42)		
**Level of education**						
Primary	310	137 (44.2)	173 (55.8)	1	1	
Post primary	434	165 (38.0)	269 (62.0)	0.86 (0.72–1.02)	0.87 (0.70–1.08)	0.208
**Duration of stay in the informal settlement**						
0–5 years	264	98 (32.5)	166 (37.6)	1	1	
6–10 years	145	74 (24.5)	71 (16.1)	1.37 (1.09–1.71)	1.34 (1.02–1.75)	0.034*
More than 10 years	335	130 (43.0)	205 (46.3)	1.05 (0.77–1.28)	0.91 (0.70–1.19)	0.521
**Drunk alcohol in the last 30 days**						
No	51	13 (4.9)	38 (11.1)	1	1	
Yes	553	303 (95.1)	250 (88.9)	1.77 (1.09–2.86)	1.46 (0.77–2.76)	0.240
**Used marijuana in the last 30 days**						
No	42	17 (8.9)	25 (10.5)	1		
Yes	386	173 (91.1)	259 (89.5)	1.10 (0.72–1.68)		
**Chewed khat in the last 30 days**						
No	41	11 (5.6)	30 (11.8)	1	1	
Yes	409	184 (94.4)	225 (88.2)	1.67 (0.99–2.81)	1.93 (1.10–3.40)	0.021*
**Used kuba in the last 30 days**						
No	624	42 (77.8)	25 (67.6)	1		
Yes	24	12 (22.2)	12 (32.4)	1.25 (0.80–1.95)		
**Used heroin in the last 30 days**						
No	13	10 (52.6)	3 (37.5)	1		
Yes	14	9 (47.4)	5 (62.5)	0.83 (0.50–1.37)		

A significance level of 0.05 was considered. CI: Confidence Interval, PR = Prevalence Ratio

## Discussion

This study among young psychoactive-substance-users, shows a high prevalence of multiple sexual partnerships in a short period of 30 days, indicating more recent sexual risk behaviour in this population. Young psychoactive-substance-users aged 18–19 years, females, those with no formal education, those living without any parent or guardian, and those earning above UGX 500,000 (≈ USD 138) reported a higher prevalence of multiple sexual partnerships compared to their counterparts. Sex, duration of staying in the informal settlement, and a history of chewing khat in the last 30 days were associated with multiple sexual partnerships, and are discussed in light of the existing literature here below.

The high prevalence of multiple sexual partnerships is not surprising given the known impact of psychoactive substance use on high-risk sexual behaviours [46,47]. This study, therefore, reinforces existing evidence about the role psychoactive substances play in shaping sexual behaviours and consequently driving the HIV epidemic. Psychoactive substance use is a key driver of multiple sexual partnerships [48], which in turn increases the risk of transmission of HIV and other sexually transmitted infections [49].

Importantly, this study points out that psychoactive substance users opt to have multiple sexual partners to satisfy their sexual desires, for pleasure, satisfaction, and economic needs. Similar findings have also been reported among young people in Ethiopia [50]. In our study, being female was significantly associated with engaging in multiple sexual partnerships. The social and economic vulnerabilities that characterise young women in informal settlements may drive them into transactional sex that is likely to result in multiple partners. Informal settlements have limited opportunities for generating income, particularly among young women and girls. As a result, these may engage in multiple sexual partnerships as a source of livelihood [18], with older persons who provide the much-needed income. Recent evidence shows that 13% of sexually active females in Kampala’s informal settlements have ever engaged in sex work [25].

The prevalence of multiple sexual partnerships was higher among young people aged 18–19 years compared to those aged 20–24 years. This high proportion may be explained by the stages of development that adolescents (18–19 years) undergo as they transition into adulthood. These stages are often characterised by experimentation and exploration [51]. As such, adolescent psychoactive substance users may be involved in sexual exploration thus a high prevalence of multiple sexual partnerships. Young people, especially females in these settings are also likely to be vulnerable to older males, taking advantage of them in periods of intoxications, and maybe more accepting of transactional sex, that yield multiple relationships [52,53].

This study also highlights the importance of staying with a parent or guardian as protective against sexual risk behaviours including multiple sexual partnerships. Young psychoactive-substance-users staying with parents may have restrictions on their sexual behaviours including who they interact with at risky periods at night and for how long. Independent Young psychoactive-substance-users may have a leeway to interact with several sexual partners at any time they wish and during periods of intoxication.

From this study, it was evident that the prevalence of multiple sexual partnerships was higher among Young psychoactive-substance-users who earned more than UGX 500,000 000 (≈ USD 138) compared to those who earned less. Young psychoactive-substance-users, particulary males, earning a higher income have the financial power to sustain multiple relationships compared to those with lower financial power. These are also more likely to pay for sex compared to those with a low monthly income. These findings are in agreement with those of a study conducted in India which indicated that men of higher economic status were more likely to engage in multiple sexual partnerships compared to those of low economic status [54]. It is also possible that income reported could have been generated from sex work especially among females, given that they also reported more multiple sexual partnerships that males. However, due to the nature of the design, we could not obtain this causal relationship.

Based on our findings, chewing khat was associated with a higher prevalence of multiple sexual partnerships. The effect of khat on high-risk sexual behaviour and particularly engagement in multiple sexual partnerships may be explained by the alcohol myopia and expectancy theories [55,56]. The use of khat is known to increase sexual desire [57]. This feeling often drives young people to seek sexual partners without evaluating the risks involved, with an impaired decision-making process. Also, psychoactive substance users may engage in multiple sexual partnerships expecting better sexual experiences (expectancies) with different partners. Similarly, Doku [7] and [58] reported that young people in Ghana and Nigeria respectively often used psychoactive substances to enhance their sexual pleasures.

### Strengths and limitations of the study

#### Strengths

This is one of the few studies that have examined the prevalence and factors associated with multiple sexual partnerships among young psychoactive-substance-users in informal settlements in low-income countries. The study used a relatively large sample size, making the results more reliable. Besides, the study used a short recall period of 30 days, thus less prone to recall bias, and indicates more recent behaviour.

#### Limitations

This study was conducted among young psychoactive-substance-users in Kampala’s informal settlements. Therefore, these findings should not be generalised to all young people in Uganda. Therefore, there is a need to examine engagement in multiple sexual partnerships among young psychoactive-substance-users in affluent and formal settlements. The cross-sectional design limits causal linkages between psychoactive substances and engaging in multiple sexual partnerships.

#### Conclusion and recommendation

This study examined the prevalence and factors associated with multiple sexual partnerships among young psychoactive-substance-users in informal settlements in Kampala, Uganda. It was found that multiple sexual partnerships are highly prevalent across different socio-demographic strata. Being female, having lived in the informal settlement for 6–10 years and chewing khat were significantly associated with having multiple sexual partners in the last 30 days. These findings are indicative of the high risk of transmission of STIs including HIV among young psychoactive-substance-users. In tackling this high-risk sexual behaviour, it is recommended that risk-reduction interventions make special emphasis across the different socio-demographic strata identified in this study, i.e. females, those who have lived in the informal settlement for about 6–10 years, and those who chew khat.

## Supporting information

S1 File Multiple sexual partnerships among young people. (XLS)

S2 File Study questionnaire. (DOCX)
