# Supplementary material for: Multiple sexual partnerships and associated factors among young psychoactive-substance-users in informal settlements in Kampala, Uganda
Source: PLoS One. 2020 Oct 6;15(10):e0239323. doi: 10.1371/journal.pone.0239323 (PMC7537871; doi:10.1371/journal.pone.0239323)
Supplement: S2 File — (DOCX) [file pone.0239323.s002.docx]

# Appendix 4: Structured Questionnaire

**Study tittle: Sexual Behaviour of Young Psychoactive Substance users in Kampala’s Informal Settlements, Uganda**

**Preamble**

Hello, my name is……………………………, I am currently working with Makerere University School of Public health as a researcher. You have been selected to participate in a survey on the ‘’**Sexual Behaviour of Young Psychoactive Substance users in Kampala’s Informal Settlements’’.** The main objective of this study is to understand the sexual behaviour of young psychoactive substance users in informal settings in Kampala. Your answers will be important for us to understand the sexual behaviour of alcohol and other substance users in informal settlements.

This is an anonymous study, and your participation is completely voluntary. We will not disclose your answers to anyone else outside our research team. We will do our best to keep your information safe by not writing down your name and using a special code on the questionnaire. We will also keep all data entries under lock and key.

It will take about 10 minutes to answer the questions. It is OK to choose “don’t know” if you don’t know the answer. If you have any questions during the survey, you may ask the study coordinator to explain/clarify. You do not have to answer all the questions and you may stop at any time. If you have any questions after the survey, you may contact Mr. Ssekamatte Tonny (0782381484; email: [ssekamattet.toca@gmail.com](mailto:ssekamattet.toca@gmail.com)). Please note that by answering the questions is an indication of voluntary participation in the study. Therefore, only respond to the questions after understanding the objectives of the study.

**Psychoactive substance user questionnaire**

In this study, I will ask you about the use of psychoactive substances such as khat, marijuana, alcohol and heroine. In addition, I will ask you questions pertaining your sexual behaviour as well as information regarding sexually transmitted diseases such as syphillis.

| **Study tittle:** Sexual Behaviour of Young Psychoactive Substance Users in Kampala’s Informal Settlements, Uganda | | | |
| --- | --- | --- | --- |
| **Identification information** | | | |
|  | Division/ **Divisoni** | 1. Makindye 2. Kawempe |  |
|  | Zone/ **Zooni** |  |  |
|  | GPS coordinates |  |  |
|  | **SECTION 1: Socio demographic characteristics of the respondents** | |  |
| **No.** | **Questions/ Ebibuuzo** | **Response/ Ebididdwamu** | **Skip** |
| 100 | Sex of the respondent**/ Ekikula** | 1. Male 2. Female |  |
|  | How old are you? (in complete years)  **Olina emyaka emmeka emijjuvu** |  |  |
|  | What is the highest level of school you attended/are in now?  **Mubyokusoma, wakoma ku ddaala ki?** | 1. No formal education 2. Primary 3. Secondary 4. Tertiary |  |
|  | Marital status  **Eby’obufumbo** | 1. Single 2. Married/ Cohabiting 3. Widowed 4. Divorced 5. Separated |  |
|  | What is your religion?  **Oli wa ddiini ki?** | 1. Catholic 2. Protestant 3. Muslim 4. Born again Christian/ Pentecostal 5. SDA 6. Other (Specify) |  |
|  | For how long have you lived in this village?  **Mukitundu kino abadde mu kumala bbanga ki?** | 1. Months **(Myezi)** 2. Years **(Myaka)** |  |
|  | What do you do for a living?  **Okola murimu ki okwebezaawo?** | 1. Fisherman 2. Fish Trader 3. Petty Trader 4. Bar Attendant 5. Peasant Farming 6. Other (Specify) |  |
|  | Roughly how much do you earn per month? This may be from sales, part time work or other work  **Bwogeregeranya ofuna ssente mmeka mumwezi?** | _______________________ |  |
|  | Are you still staying with your parents? | 1. Yes 2. No |  |

**Section two: History of psychoactive substance use**

The next few questions are about the use of PSYCHOACTIVE SUBSTANCES such as Beer, Waragi, Tonto, khat, marijuana, heroin and others by yourself and by people you know.

**Ebibuuzo ebiddako by’ekuusa kukukozesa ebitamiiza nga ‘’beer’’, omunanaansi, tonto, waragi, amalwa ebiragalalagala nga enjaga, amairungi n’amafuta g’enyonyi.**

READ TO RESPONDENT: I am going to read to you some of the psychoactive substances commonly used in Uganda and I request you tell me whether you have ever used them or currently using them.

**Instruction: If the answer to the questions below is ‘NO’ write ‘NA’ for “not applicable” for next question then ask about the next method**

| No. | Drug/ substance  **Ekiragalalagala** | Ever used?  **Wali Okozesezaako?** | Used drug/ substance in the last 12 months?  **Okozesezaako mu myezi 12 egiyise?** | Have you used it in the last 30 days?  **Okozesezaako mu nnaku 30 eziyise?** | During the **last 30 days**, how  often did you use any of those substances  **Munnaku asatu eziyise, mirundi emmeka gy’okozesa omwenge oba ebiragala?** | Are there times when any of the members of your social network (Friend, relatives, other) has condoned you to drink or to use this substance? **Wali wabaddewo akaseera nga mikwano gyo egy’okulusegere negikugaana okunywa omwenge oba okozesa ebiragalalagala?** | |
| --- | --- | --- | --- | --- | --- | --- | --- |
|  |  |  |  |  |  |  | if yes, by whom?  indicate whether (spouse, children, friends or other) |
|  | Alcohol (Wines, spirits and beer) (omwenge/ waragi) | yes……...1  No……...2 | yes……...1  No……...2  NA……...3 | yes……...1  No……...2  NA……...3 | 1. Monthly or less 2. 2 to 4 times a month 3. 2 to 3 times a week 4. 4 or more times a week 5. N/A | yes……...1  No……...2  NA……...3 |  |
|  | Cannabis **(Njaga/ sada)** | yes……...1  No……...2 | yes……...1  No……...2  NA……...3 | yes……...1  No……...2  NA……...3 | 1. Monthly or less 2. 2 to 4 times a month 3. 2 to 3 times a week 4. 4 or more times a week 5. N/A | yes……...1  No……...2  NA……...3 |  |
|  | Khat **(Mairungi)** | yes……...1  No……...2 | yes……...1  No……...2  NA……...3 | yes……...1  No……...2  NA……...3 | 1. Monthly or less 2. 2 to 4 times a month 3. 2 to 3 times a week 4. 4 or more times a week 5. N/A | yes……...1  No……...2  NA……...3 |  |
|  | Kuba | yes……...1  No……...2 | yes……...1  No……...2  NA……...3 | yes……...1  No……...2  NA……...3 | 1. Monthly or less 2. 2 to 4 times a month 3. 2 to 3 times a week 4. 4 or more times a week 5. N/A | yes……...1  No……...2  NA……...3 |  |
|  | Heroin **(Mafuta g’enyonyi)** | yes……...1  No……...2 | yes……...1  No……...2  NA……...3 | yes……...1  No……...2  NA……...3 | 1. Monthly or less 2. 2 to 4 times a month 3. 2 to 3 times a week 4. 4 or more times a week 5. N/A | yes……...1  No……...2  NA……...3 |  |
|  | **Kabanga** (mixture of tobacco and cannabis) | yes……...1  No……...2 | yes……...1  No……...2  NA……...3 | yes……...1  No……...2  NA……...3 | 1. Monthly or less 2. 2 to 4 times a month 3. 2 to 3 times a week 4. 4 or more times a week 5. N/A | yes……...1  No……...2  NA……...3 |  |
|  | Other substance (Record the substance) | yes……...1  No……...2 | yes……...1  No……...2  NA……...3 | yes……...1  No……...2  NA……...3 | 1. Monthly or less 2. 2 to 4 times a month 3. 2 to 3 times a week 4. 4 or more times a week 5. N/A | yes……...1  No……...2  NA……...3 |  |

**Information on sexual behaviour**

Now, I want to ask you questions pertaining your sexual behaviour. Some of these questions may be sensitive but I would like to assure you that all the responses you give me will be treated with utmost confidentiality. I promise you that no one will get to know whatever we discuss. You may feel free not to answer those questions that may negatively impact on your mental health. However, answering those questions will be useful in thoroughly understanding the sexual behaviour of people who use psychoactive substances. Can I proceed with the interview? **If yes, proceed with the interview and if No, terminate the interview**

| **No.** | **Question** | **Responses** | **Skip** |
| --- | --- | --- | --- |
|  | Have you ever had any sexual intercourse?  **Wali wegaseko muby’omukwano?** | 1. Yes 2. No |  |
|  | How old were you when you first had sexual intercourse?  **Walina emyaka emmeka wewasokera ddala okwegatta muby’omukwano?** | Age _______________  **Emyaka** |  |
|  | Are there occassions when you had sexual intercourse with somebody when you are under the influence of alcohol or had taken alcohol?  **Wali wabaddewo akaseera wewegatta n’omuntu nga osindikiribwa omwenge oba ebiragalalagala?** | 1. Never had any sexual Partner 2. Never happened 3. Rarely 4. Often 5. Always 6. Declined to answer |  |
|  | How many times have you had sex in past one month?  **Mirundi emmeka gy’ewegasse mu mwezi oguwedde?** | 1. Times **(Emirundi)** 2. Can’t remember |  |
|  | How many sexual partners did you have in the past one month?  **Mumwezi oguyise wegasse n’abantu bammeka?** | 1. Partners 2. Can’t Remember |  |
|  | How many sexual partners in past 12 months?  **Atte mu myezi 12 egiyise, weggase n;abantu bammeka?** | 1. Partners 2. Can’t Remember |  |
|  | What main reason leads you to having more than one sexual- partner?  **Ki ekisinga okuletera okubeera n’abantu abokwegata nabo abangi?** |  |  |
| **Recall of details for each recent relationship (past 12 months)**  **Nsaba ojjukire abantu abo bonna bewegatta nabo mu myezi 12 egiyise** | | | |
|  | MEN: What is your relationship to the woman with whom you last had sex?  WOMEN: What is your relationship to the man with whom you last had sex?  **ABASAJJA: Wayina nkolagana ki n’omukazzi gwewasembayo okwegatta naye?**  **Wayina nkolagana ki n’omusajja gwewasembayo okwegatta naye?** | 1. Spouse 2. Boy/Girl Friend 3. Another Friend 4. Casual Acquaintance 5. Commercial Sex Worker 6. Other (Specify) |  |
|  | The last time you had sexual intercourse, did you or your  Partner drink alcohol before sex? IF YES: Who used such as substance?  **Omulundi gwewasembayo okwegatta kugwe oba omwagalwawo wali okozesezaako ku kitamiiza oba ebiragalalagala?**  **Oba Ye, ani?** | 1. Respondent Only 2. Partner Only 3. Respondent and Partner 4. Neither 5. Declined to respond |  |
|  | Were you or partner drunk or used any other substance before last sex?  **Wemwegattira, gwe oba omwagalwawo kwaliko atamidde oba waliwo eyali akozeseza ebiragalalagala?** | 1. Respondent Only 2. Partner Only 3. Respondent and Partner 4. Neither 5. Declined to respond |  |
|  | For how long have you had sexual relations with this partner?  **Omazze bbanga ki ngawegatta n’omuntu oyo?** | \|  \|  \| \| --- \| --- \| \|  \|  \| \|  \|  \| \|  \|  \|   Days  Weeks  Months  Years |  |
|  | How do you describe the level of condom use with this partner during the period of the relationship?  **Mwakozesa kondomu bulikiseera oba nedda?** | 1. All the time 2. Only sometime 3. Never |  |
|  | What was the main reason for not using a condom or inconsistently using it?  **Nsonga ki eyabalemesa okukozesa kondomu obudde bwona?** | 1. Respondent wanted a child 2. Trusted partner 3. Partner insisted not to use it 4. Other 5. Don't Know |  |
|  | Where did you get the condom for the last sex with this person?  **Kondomu wagigyawa omulundi gwe wegatta n’omuntu ono?** | 1. Shop 2. Clinic/Drug Shop 3. Market 4. I Don’t Know. 5. He/ She Had It 6. Other (Specify) |  |
|  | How long was it between first meeting that person and first having sex with the partner?  **Kyakutwalira bbanga ki okuva wewasanga omuntu ono n’okwegatta naye?** | 1. Within 24 hours 2. Between 1 day and 1 week 3. Between 1 week and 4 weeks 4. Between 4 weeks and 6 months 5. Between 6 Months and 1 year 6. More Than 1 Year |  |
|  | How did you meet your latest partner?  **Wasisinkana otya omuntu gwewasembanaye okwegatta?** | 1. Church 2. Through friends/relatives 3. At school work 4. Social event organized 5. Through a friend 6. Society, club or, interest group 7. Neighbours /Family friends 8. Others (Specify) |  |
|  | **Perceptions on the influence of psychoactive substance use on sexual behaviour** | |  |
|  | Do you think alcohol or psycho active substance use improves your sexual performance? **Olowooza omwenge oba ebiragalalagala bikwonegeramu ammanyi mu by’okwegatta?** | 1. Yes 2. No |  |
|  | When drinking or using other substances, how often do you think about sex?  **Bw’oba onnywa omwenge oba nga okozesa ebiragalalagala (erinnya okugeza njaga), mirundi emmeka gy’olowooza ku kwegatta?** | 1. Always 2. Sometimes 3. Uncertain 4. Never |  |
|  | Have you ever got a woman/man drunk/ given him/her drugs in order to have sex? **Wali otamiziiza ko omuntu kubanga oba okumuwa ebiragalalagala nga oyagala mwegatte mumukwano?** | 1. Yes 2. No |  |
|  | Do you use a drinking/ drug use establishment as a place to meet sexual partners? **Otera okukozesa ebifo ebinywerwamu omwenge oba ebiragalalagala okusisinkana abagalwabo?** | 1. Yes 2. No |  |
|  | How often have you found it difficult to use a condom when you were drunk or had used drugs?  **Mirundi emmeka gy’ofuna obuzibu okukozesa obupiira ng’otamidde oba nga okozeseza ebiragalalagala?** | 1. Never 2. Once only 3. Occasionally 4. Often 5. Always |  |
|  | If you have ever found it difficult to use a condom when you were drunk, how often has this happened with a sex worker?  **Bw’oba ofuna obuzibu okukozesa obupiira nga okozeseza ebiragalalagala, kino ky’akabaawo emirundi emmeka?** | 1. Never 2. Once only 3. Occasionally 4. Often 5. Always |  |
|  | Are you more likely to engage in sex when under the influence of psychoactive substancs?  **Otera okwagala okwegatta nga okozeseza ebiragalalagala?** | 1. Yes 2. No |  |
|  | Is sex more pleasurable when under the influence of psychoactive substances?  **Okwegatta kusinga kunyumira nga okozeseza ebiragalalagala?** | 1. Yes 2. No |  |
|  | Does being under the influence of psychoactive substances make it difficult for you to use condoms?  **Bw’oba okozeseza ebiragalalagala kitera okuzibuwalira okukozesa obupiira?** | 1. Yes 2. No |  |
|  | Does being under the influence of psychoactive substances make you forget to use a condom?  **Bw’oba nga onywedde omwenge oba ebiragalalagala, kitera okuretera okwerabira okozesa obupiira?** | 1. Yes 2. No |  |
|  | Do you find it difficult to refuse sex when under the influence of alcohol or substances?  **Otera okukisanga mu obuzibu okugaana okwegatta nga okozeseza omwenge oba ebiragalalagala?** | 1. Yes 2. No |  |
|  | Do you use psychoactive substances to give yourself courage to approach a partner for sex?  **Otera okunnywamu okufuna obuvumu okusaba omuntu akaboozi?** | 1. Yes 2. No |  |
|  | Do you find yourself wanting to have sex when using psychoactive drugs?  **Otera okwagala okwegatta nga okozeseza ebiragalalagala?** | 1. Yes 2. No |  |
|  | Have you ever had sex with a commercial sex worker?  **Wali wegaseko n’omuntu atunda akaboozi?** | 1. Yes 2. No |  |
|  | Have you ever had drunk sex with a commercial sex worker?  **Wali wegasse ko n’atunda akaboozi ng’a otamidde oba nga okozeseza ebiragalalagala?** | 1. Yes 2. No |  |
|  | Have you ever used a condom when having drunk sex with a commercial sex worker?  **Wali okozesezako kondomu nga wegatta ne nneeko?** | 1. Yes 2. No |  |
|  | Did you use a condom the last time you had sex with a commercial sex worker?  **Wakozesa kondomu wewasembayo okwegatta ne nneeko?** | 1. Yes 2. No |  |
|  | What was the main reason for not using a condom or inconsistently using it?  **Nsonga ki eyabalemesa okukozesa kondomu obudde bwona** | 1. Respondent wanted a child 2. Trusted partner 3. Partner insisted not to use it 4. Other (specify) 5. Don't Know |  |
|  | Have you had a non-spouse sexual partner in the past 30 days? ***(Besides a commercial sex worker)***  **Nga ogyeko Nneeko, wali wegaseko n’omuntu omulala atali mukyala wo mu by’omukwano?** | 1. Yes 2. No |  |
|  | The last time you had sex with a non-spouse partner, did you use condoms?  **Wewasembayo okwegatta n’omuntu oyo, wakozesa akapiira?** | 1. Yes 2. No |  |
|  | What was the main reason for not using a condom or inconsistently using it?  **Nsonga ki eyabalemesa okukozesa kondomu obudde bwona** | 1. Respondent wanted a child 2. Trusted partner 3. Partner insisted not to use it 4. Other (specify) 5. Don't Know |  |
|  | **Information on sexually transmitted diseases** | |  |
|  | Have you ever heard of any sexually transmitted diseases?  **Wali owuliddeko ku ndwadde z’obukaba?** | 1. Yes 2. No |  |
|  | What sexually transmitted diseases do you know? *Tick all that apply*  **Ndwadde ki z’ewali owuliddeko?** | 1. HIV/AIDS 2. Syphilis 3. Gonorrhea 4. Chlamydia 5. Genital warts 6. Hepatitis B 7. Herpes simplex 8. Other (specify) |  |
|  | Have you suffered from any of these diseases in the last 12 months?  **Olwadde ko obulwadde buno mumyezi 12 egiyise?** | \| **Disease** \| **Y** \| **N** \| \| --- \| --- \| --- \| \| HIV/AIDS \|  \|  \| \| Syphilis \|  \|  \| \| Gonorrhea \|  \|  \| \| Chlamydia \|  \|  \| \| Genital warts \|  \|  \| \| Hepatitis B \|  \|  \| \| Herpes simplex \|  \|  \| |  |
|  | Have you ever tested for any of the following STIs?  **Wali wekebezesako ku ndwadde zino wamanga?** | \| **Disease** \| **Y** \| **N** \| \| --- \| --- \| --- \| \| HIV/AIDS \|  \|  \| \| Syphilis \|  \|  \| \| Gonorrhea \|  \|  \| \| Chlamydia \|  \|  \| \| Genital warts \|  \|  \| \| Hepatitis B \|  \|  \| |  |
|  | If yes where did you test?  **Oba ye, wekeberezawa?**  Place for the test can be; Health unit, Clinic, Outreach program or Other (specify) | \| **Disease** \| **Place for test** \| **Y** \| **N** \|  \| \| --- \| --- \| --- \| --- \| --- \| \| HIV/AIDS \|  \|  \|  \|  \| \| Syphilis \|  \|  \|  \|  \| \| Gonorrhea \|  \|  \|  \|  \| \| Chlamydia \|  \|  \|  \|  \| \| Genital warts \|  \|  \|  \|  \| \| Hepatitis B \|  \|  \|  \|  \| \|  \|  \|  \|  \|  \| |  |
|  | If No, why? |  |  |

**Thank you very much for taking time to answer these questions.**
